# Developing a Strategy for Interventional Molecular Imaging of Oxidized Low-Density Lipoprotein in Atherosclerosis

**DOI:** 10.1177/1536012117723788

**Published:** 2017-09-06

**Authors:** Samata S. Pandey, Dorian O. Haskard, Ramzi Y. Khamis

**Affiliations:** 1 National Heart and Lung Institute, Imperial College London, London, UK

**Keywords:** molecular imaging, atherosclerosis, near-infrared fluorescence, oxLDL, autoantibody, vulnerable plaque

## Abstract

The identification of vulnerable coronary artery atherosclerotic plaques offers the prospect of either localized or systematic therapeutic targeting in order to prevent myocardial infarction. Molecular imaging of atherosclerosis adds to morphological imaging by focusing on the immunobiology hidden in and behind the endothelium and therefore may be able to improve the identification of prospective culprit lesions. Our focus has been on identifying arterial accumulation of oxidized low-density lipoprotein (oxLDL) by exploiting advances in knowledge of vascular pathobiology. Here, we reflect on our work developing near-infrared fluorescence imaging of oxLDL using LO1, a monoclonal autoantibody generated in our laboratory. We detail progress to date and discuss our vision on taking the work through the early translational pipeline toward a multitargeted approach in imaging rupture-prone atherosclerotic plaques. Ultimately, molecular imaging of coronary arteries should be able to assess the regional risk that is specific to a lesion, which can then be used in concert with global risk factors to personalize the therapeutic strategy for patients in a way that goes beyond generalized population-based therapies.

Coronary heart disease is the UK’s single biggest killer, being responsible for nearly 70 000 deaths in the UK each year.^1^ Approximately 20% of patients with acute coronary syndromes that have been successfully treated with stents as well as contemporary optimal medical therapy develop major adverse cardiovascular events within 3 years.^2^ It remains clinically challenging to identify which atherosclerotic plaques are destined to rupture and cause clinical events.^3^ Intravascular imaging techniques such intravascular ultrasound (IVUS) and optical coherence tomography (OCT) and noninvasive techniques such as coronary artery computed tomography (CT) and coronary magnetic resonance imaging have made considerable progress in defining atherosclerotic plaque morphology.^4,5^ Furthermore, the ability of morphological analysis to predict plaque progression can be augmented by identifying local regions of low endothelial shear stress.^6^ However, even in combination, the ability of these techniques to identify plaques that will rupture remains limited.^6^

Significant advances have already been achieved toward the molecular imaging of atherosclerosis.^7^ Near-infrared fluorescence (NIRF) optical molecular imaging offers a new approach to the evaluation of coronary plaque biology, especially helped by the development of intravascular NIRF detectors.^8,9^ Compared to the use of radionuclear reporters, intravascular NIRF targeting would have the advantage of not adding radiation as well as providing better spatial resolution compared to whole-body approaches. The ultimate goal of NIRF molecular targeting is to integrate specific multi-agent biological information with morphological imaging obtained by existing catheter-based technologies (ie, IVUS and OCT), thereby providing a much needed extra dimension for improving the prediction of individual plaque progression and instability.^10,11^

Fluorescence molecular tomography (FMT) is a powerful NIRF preclinical modality that allows simultaneous quantitative molecular imaging in small animals of one or more tracers labeled with dyes operating in distinct near infra-red spectra.^9,12,13^ When combined with CT, FMT has revolutionized the ease with which molecular targets may be studied, offering a nonradioactive near equivalent to single-photon emission CT and positron emission tomography.^14^

Oxidized LDL (oxLDL) is a prime target for the molecular imaging of atherosclerosis, not only because it is instrumental in plaque pathobiology but also because its presence may reflect plaque vulnerability.^15,16^ Imaging of oxLDL in atherosclerosis has already been reported in preclinical ex vivo studies using anti-oxLDL antibodies conjugated to single-photon emitting isotopes,^17,18^ and also in vivo with anti-oxLDL-labeled MR contrast agents.^19,20^ As yet, we are the only group to report quantitative optical molecular imaging of oxLDL in atherosclerosis in conjunction with other molecular probes.^9^

Our laboratory isolated and characterized LO1, a spontaneously arising IgG3k germ line encoded mouse natural monoclonal autoantibody. It was selected for reacting with copper-oxidized LDL in vitro and subsequently found to recognize LDL conjugated with malondialdehyde. LO1 was shown to bind antigen in atherosclerosis by in vitro staining of both mouse and human tissues.^21^ We labeled LO1 with the NIRF dye VivoTag-S 750 (and designated this LO1-750), and this specifically reacted with determinants in the necrotic core in human coronary lesions ex vivo.^9^ Injection of LO1-750 into high-fat (HF)-fed atherosclerotic *Ldlr^−/−^* mice led to specific focal localization within the aortic arch and its branches, as detected by FMT combined with micro-CT. Ex vivo confocal microscopy confirmed LO1-750 subendothelial localization at sites of atherosclerosis, in the vicinity of macrophages. When compared with an NIRF reporter of metalloprotease (MMP) activity (MMPSense-645-FAST), both probes produced statistically significant increases in NIRF signal in the *Ldlr^−/−^* model in relation to duration of HF diet, with a better target to background ratio (TBR) for LO1-750 (LO1-750 19.8; MMPSense 2.8). The ability of LO1-750 to quantify atherosclerotic load was further tested in a progression study in the *Ldlr^−/−^* model. LO1-750 clearly distinguished between a group fed a HF diet for 42 weeks and a group fed for just 30 weeks. We also studied a middle group that was fed for 30 weeks with a HF diet, and low fat diet for 12 further weeks, and found a correlation trend (*R* ^2^ = .42, *P* < .05) between all groups. MMPSense was also able to distinguish between the 2 feeding extremes of the experiment but had a lower correlation trend, indicating that the middle group was not as clearly distinguished as with LO1. The differential identification of the middle group by LO1 is consistent with reporting on the accumulation of the antigen it identifies in plaque, namely, oxLDL. MMPSense, however, tracks MMP activity, and stopping the pro-inflammatory stimulus from the HF diet “turned off” the progression of the MMP signal in the intermediate group. In due course, it may therefore be possible to use both agents simultaneously to distinguish patients between plaque inflammation (ie, MMP activity) and plaque progression (ie, oxLDL content). For example, it may be that a particular treatment reduces inflammation (and MMP activity) and the risk of plaque rupture in the short term but fails to halt the buildup of oxLDL (and LO1 reactivity) and clinical manifestations in the longer term.

As a proof of concept that NIRF imaging with LO1 is applicable to a catheter-based approach, we undertook pilot studies in the laboratory of our collaborator Farouc Jaffer at Harvard Medical School using a 2-D NIRF system combined with IVUS. This system has been successfully used to image balloon injury and stent-induced inflammation in a HF-fed rabbit model.^22-24^ The imaging of intravenously (IV) injected LO1-750 with 2-D NIRF in the live animal was suboptimal and did not reflect the whole area identified later with ex vivo fluorescence reflectance imaging. This was likely due to a high LO1-750 background signal remaining in blood, as well as suboptimal tissue penetration in the rabbit, despite imaging being undertaken 21 hours postinjection. Interestingly, using ex vivo intravascular 2-D NIRF imaging, with the aorta flushed completely empty of blood and perfused with saline, we did clearly demonstrate that IV injected LO1-750 could be detected in the diseased area identified on IVUS with a signal to noise ratio of 86.4 and a TBR of 4.8. This was confirmed by matching whole aorta fluorescence reflectance imaging ex vivo with the lesional areas identified by LO1 intravascularly. Importantly, we also demonstrated that the LO1-750 signal was different to the autofluorescence detected in the FITC channel. Furthermore, ex vivo fluorescence microscopy studies of freshly frozen sections showed a pattern of staining in the rabbit atherosclerosis model similar to that seen in LO1-750-injected mice.^9^

Although LO1 has shown great promise in targeting oxLDL within plaques in mouse and rabbit experimental models and in vitro in human, there are obvious problems in using a native mouse IgG clinically. These include suboptimal pharmacokinetics, Fc-related effector function, and immunogenicity, all of which can be improved by creating recombinant molecular derivatives of the parent antibody. With this in mind, we have successfully sequenced and cloned the LO1 VH and VL chain regions, allowing us to molecularly express the antibody in different formats. As a start, we have created a molecularly expressed human chimeric LO1 Fab construct (human CH/CL and LO1 VH/VL) with a cysteine tag (for labeling) and shown that its function resembles that of LO1 in vitro.^9^ Moreover, our initial pilot data in vivo in mice show that LO1-Fab-Cys targets atherosclerotic lesions in *Ldlr^−/−^* mice in a similar manner to LO1 native IgG.*^9^*

To further improve our construct, fragments such as single-chain variable fragment and diabodies will be explored to achieve the optimal avidity, penetrance, and background clearance. However, it must be noted that due to the chimeric nature of LO1-Fab, repeated administrations may elicit a human antimouse immune response and thus more fully humanized constructs will also be generated.

Bringing an NIRF molecular imaging tool into interventional cardiology will hopefully not only help management in the cardiac catheter laboratory but also provide a platform for the application of anti-oxLDL antibodies and other agents for therapeutic “pay load” delivery. We envisage the development of oxLDL targeting to go hand-in-hand with other targets toward a multipronged approach for the targeting of rupture prone plaques in atherosclerosis, which is still one of the biggest causes of mortality and morbidity in the world.
